# Touch Supports Word Segmentation More Than Statistics But is Not Mediated by Attention

**DOI:** 10.1111/infa.70123

**Published:** 2026-08-27

**Authors:** Amanda Seidl, Boshang Yin, Bridgette Kelleher

**Affiliations:** ^1^ Department of Communication Sciences and Disorders University of Delaware Newark Delaware USA; ^2^ Department of Psychological Sciences Purdue University West Lafayette Indiana USA

**Keywords:** attention, heart rate, infants, word segmentation

## Abstract

Multisensory information is integral to infants' language experiences and research has suggested that infants can use multisensory cues to support their segmentation of wordforms continuous speech. However, the role of multisensory cues in wordform segmentation compared to that of cues present in the acoustic stream alone (e.g., transitional probability) remains unclear. In the current study, we investigated word segmentation with aligned tactile cues in 8.5‐month‐old infants to explore how these infants weight tactile cues compared to, as well as in combination with, transitional probability cues. Aligned tactile cues had a significant impact on wordform segmentation and recognition performance as assessed through orientation time in Headturn Preference Procedure, while transitional probability cues did not have a significant impact. We also explored whether performance was related to attention as measured by heart rate defined sustained attention (HRDSA). Overall, duration of sustained attention assessed through HRDSA was associated with overall longer orientation times regardless of stimulus type, but HRDSA did not vary significantly with tactile cue presence/absence or transitional probabilities.

## 1 Introduction

Infants receive rich streams of multisensory information accompanying the speech that they hear via their auditory input from caregivers. For example, a caregiver may talk to, smile at, gesture to, and touch their baby all while feeding them. These rich streams of multisensory information may serve to support infants' ability to find and segment wordforms from the continuous speech that marks the majority of their input (Johnson et al. 2014; Swingley 2005; Van de Weijer 1998) and is an important prerequisite for word learning (e.g., Estes et al. 2007). While there is no single reliable cue that infants can use to segment the speech stream into individual wordforms (even in infant‐directed speech; Van de Weijer 1998), infants have been shown to make use a multitude of cues in their auditory input to segment continuous speech into wordforms (e.g., transitional probabilities between syllables, Saffran et al. 1996; allophonic cues, Jusczyk et al. 1999; or prosodic cues, Johnson and Jusczyk 2001) that may appear in their protolexicons (e.g., Ngon et al. 2013). One such useful cue, present in the input, is statistics such as the transitional probabilities (TPs) between syllables. TPs refer to the probability that an infant will hear syllable X if syllable Y also occurs (calculated by dividing the frequency of XY by the frequency of X). Specifically, results from infants show (e.g., Saffran et al. 1996; Thiessen and Saffran 2003) that after listening to a brief Familiarization of continuous speech with no other cues to word edges, infants are able to differentiate via listening time to wordforms with sequences of syllables which always occurred together (e.g., syllable “bah” always appears adjacent to “gah”; 100%) versus ones that occurred together less frequently (e.g., the transitional probability between syllables is 33%). For example, Pelucchi et al. (2009), following Saffran et al. (1996), showed that English‐learning infants are able to track the transitional probabilities between syllables in an unknown language–here Italian input and use this knowledge to segment wordforms from this unfamiliar language. Specifically, this tracking of transitional probabilities led English‐learning infants to extract high transitional probability items better than low transitional probability items from a stream of continuous Italian speech despite a lack of familiarity with Italian.

In addition to the myriad of cues in the speech stream itself, accompanying multisensory information, when well‐aligned with word boundaries, may also serve to support infants' word segmentation. For example, Hollich et al. (2005) found that 7.5‐month‐olds were better able to segment wordforms from speech in noise when it was paired with accompanying, and well‐aligned, visual cues. Similarly, Seidl et al. (2015) found that 5‐month‐olds were better able to segment wordforms from a made‐up language with no clear acoustic cues to word edges when wordforms were accompanied by consistent and well‐aligned tactile cues. In sum, it appears that multisensory cues, when consistent and well‐aligned with speech, support, rather than distract, from infants' developing wordform segmentation skills.

However, we do not know how useful these cues are in comparison to cues present in the acoustic stream. Specifically, in the two multisensory segmentation studies cited above (Hollich et al. 2005; Seidl et al. 2015), tactile and visual cues provided *supportive* information, but were not pitted against other sources of information that we know infants use to segment speech, such as the transitional probabilities between spoken syllables. Thus, we do not know whether infants weight transitional probability cues in the auditory stream more or less heavily than cues *not* present in the auditory stream (e.g., tactile cues). Here, we asked whether accompanying and well‐aligned tactile cues which occur alongside speech are weighted more or less heavily than transitional probability cues in word segmentation and also how touch during a word segmentation task might impact infants' attentional patterns. Our goals were twofold: First, we wanted to discern whether a non‐auditory and highly social cue, like touch, could be as heavily weighted, in a linguistic task like word segmentation, as a cue present in the speech stream itself. Second, since previous studies have shown that touch does support word segmentation, we wanted to gain a greater understanding of *how* touch might support segmentation and whether effects of touch were driven by attention to this cue.

Tactile cues are *socially* relevant signals which are pervasive in early development. It has been established that young infants receive tactile cues at a high frequency in social contexts (e.g., caregiver‐infant interactions; Feldman et al. 2010; Jean et al. 2009; Stack and Muir 1990). Further, they are important signals that help to regulate infants' arousal and reduce distress which can have long‐term effects on later development. For example, touch has been shown to reduce infant distress during the still‐face paradigm both behaviorally and physiologically (Stack and Muir 1990; Stack and Muir 1992; Feldman et al. 2010) and to exert long‐term effects on cognitive development such that infants who are provided with early NICU skin‐to‐skin contact show better cognitive outcomes as much as 10 years later (Feldman et al. 2014). In typical development, despite cultural variation (e.g., Richman et al. 1988 shows double the time in tactile contact to Kenyan vs. American infants), tactile signals are highly frequent signals provided by caregivers to very young infants, though this decreases across development (e.g., Ferber et al. 2008). The tactile signal also appears, in the input, to be well‐aligned with both spoken word and phrase boundaries. For example, both Ko et al. 2023; Abu‐Zhaya et al. 2017 recorded and annotated observations of American and Korean caregivers interacting in naturalistic situations with their young infants. Analyses from these studies reveal that the majority of caregiver touches to infants were well‐aligned (within 500 ms) with words. Further, Kadlaskar et al. (2019) showed that infants were more responsive to caregivers when speech and tactile cues appeared in combination. Thus, in total, this work suggests that touches are socially relevant, frequent, *and* informative to spoken language constituency in the input to young infants. Since such signals are frequent, informative, and important to infants socially and cognitively, it is useful to ask what effect they may have on important language learning tasks such word segmentation.

It is possible that touch, when well‐aligned with word edges, aids in word segmentation for infants because (1) it is a socially relevant signal that adds information about constituent edges and/or (2) it heightens sustained attention to constituent edges. Specifically, following (2), we might hypothesize that touch induces sustained attention, which in turn leads to better processing of acoustic input. It is possible to measure sustained attention physiologically by monitoring infant heart rate (heart rate defined sustained attention; HRDSA). We suspect that touch might induce greater levels of HRDSA because previous work reveals both greater amounts of HRDSA to social stimuli (e.g., Bradshaw et al. 2024) and greater sustained attention to stimuli with accompanying touch in infants (Longa et al. 2021). That said, even if touch plays a critical role in infant word segmentation, it is possible that attention does not mediate this role. Instead, it is possible that the role touch plays in word segmentation is simply that of adding extra aligned information to the accompanying stream of speech. Specifically, tactile information could support word segmentation by simply adding *redundant information about constituent edges*. The Intersensory Redundancy hypothesis (L. E. Bahrick and Lickliter 2000; L. Bahrick and Lickliter 2002) would support this alternative. For example, in L. E. Bahrick and Lickliter (2000), 5‐month‐old infants showed better discrimination of hammer tap patterns that occurred with auditory and visual synchronicity than those which were unimodal—suggesting that an additional amodal informational stream was essential to discrimination of patterns. Note that these two hypotheses—attention‐based or intersensory redundancy—are not mutually exclusive, and it could be that both serve to support better segmentation of wordforms aligned with touches. Nonetheless, to better understand what mechanism(s) supports the role that aligned touch plays in word segmentation, we explored whether attentional patterns changed as a result of touch and, if so, whether these changes in attentional patterns were related to infant behavioral preferences. In order to examine both word segmentation with aligned tactile cues and how infants weight these tactile cues compared to, and in combination with, transitional probability cues, we utilized the same protocol as the aforementioned Pelucchi et al.’s Experiment 3A/B (thanks to their generous sharing of their stimuli). Pelucchi et al. (2009) familiarized infants to ∼2 min of Italian speech which contained 2 high (HTP) and 2 low transitional probability (LTP) words and then used the Headturn Preference Procedure (HPP; Kemler Nelson et al. 1995) to examine whether infants showed differential orientation times to isolated HTP and LTP wordforms as an index of segmentation ability; the original Pelucchi study also examined non‐words. Their results revealed that HTP words were better segmented than LTP words. We used the same speech Familiarization and Test words in Pelucchi et al. (2009) but further manipulated their ∼2 min input stream so that *one* of each of their two HTP and LTP wordforms was accompanied by consistent well‐aligned experimenter‐delivered touches. In contrast, the other HTP and LTP wordform was not accompanied by any touches. Infants were then tested on their looking time preferences to isolated HTP‐Touch, LTP‐Touch, HTP‐NoTouch, and LTP‐NoTouch wordforms in the HPP.

In addition, during both Familiarization and Test, infants wore a heart rate monitor which allowed us to examine heart rate defined sustained attention (HRDSA) in both experimental phases and how HRDSA related to behavioral preference patterns. Thus, this design allowed us to examine how aligned and consistent tactile cues interacted with infants' use of transitional probability cues during segmentation of a novel Italian speech stream and whether sustained attention was impacted by aligned touch cues. We predicted that, as in Pelucchi et al. (2009), infants would show longer orientation times to HTP over LTP wordforms and further, that touch would have an additive effect, such that HTP wordforms with aligned touch would show longer orientation times over HTP wordforms without touch. Further, we predicted that behavioral patterns would be related to patterns of sustained attention and that increased HRDSA during touches may drive behavioral results.

## 2 Methods

### 2.1 Participants

Caregiver‐infant dyads were recruited from Purdue University, via Facebook, University participant registries, local WIC center recruitment tables, as well as tabling at a variety of other local events. The study was conducted in accordance with the ethical standards of the American Psychological Association and was approved by the IRBs of the University of Delaware AND Purdue University [Protocol number 2079099 and IRB‐2022‐1471]. If caregivers registered interest via any of these mechanisms, lab staff contacted them via phone or text with further information about the study. If they responded with interest in participating, they were scheduled for a single visit. Consent was obtained from all caregivers who participated in the current study. A total of 56 infants were tested. Nineteen infants were excluded due to fussiness, experimenter error, and/or equipment failure. The final sample included 37 infants (16 female, 21 male) with a mean age of 8.48 months (range = 6.28–11.1 months). 86.5% of infants were identified by caregivers as White, 2.70% were identified as Asian, 8.11% were identified as Multiracial, and 2.70% were identified as other races. 16.2% infants were identified by caregivers as Hispanic. All infants came from a community of ∼44,000 people in which the median income was ∼$34,000. Power was evaluated for these sample sizes for standardized beta coefficients from a multiple regression model with 4 covariates, ***α*** = 0.05, power (1−*β*) greater than or equal to 0.80, 15% attrition, and nesting of repeated measures (ICC) = 0.30. Effective sample sizes based on these specifications (attrition, ICC, and number of repeated measures) were used to determine power. The major hypotheses involve within‐subject tests, which are more powerful with higher ICC, i.e., higher between‐subject variance. In addition, we based our sample size on the Pelucchi et al. (2009) study as well as additional studies with a similar design which reveal a moderate effect size with samples from 24 to 35 participants (e.g., see a meta‐analysis in Isbilen and Christiansen 2022).

### 2.2 Equipment

**FIGURE 1:**
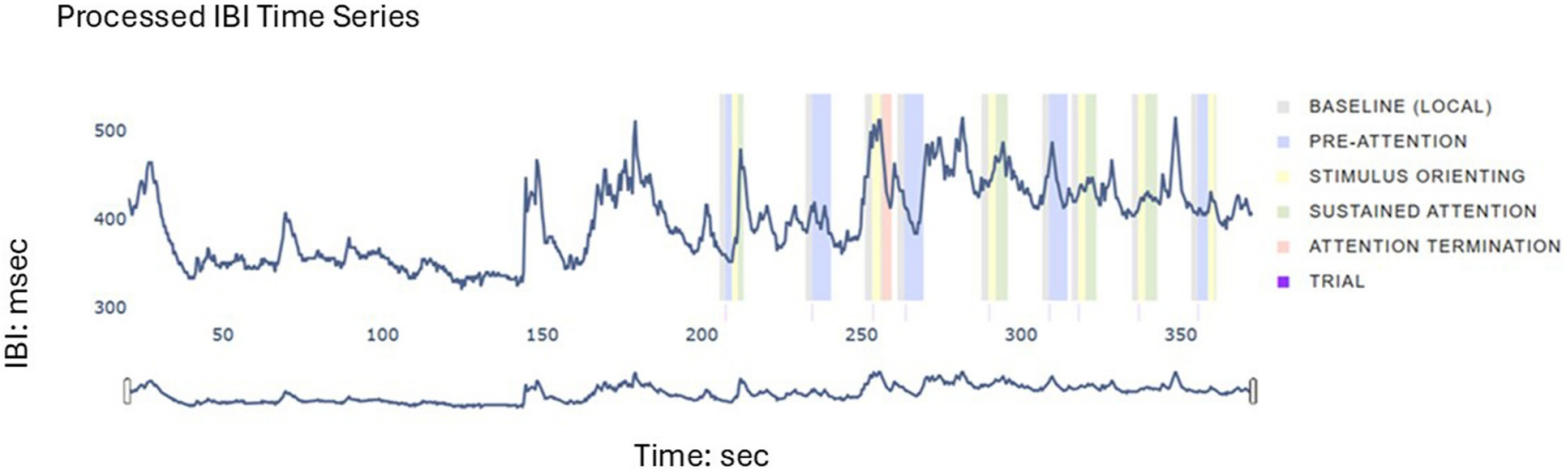
Example of the IBI identification viewer in haven.

A Bittium Faros 180 ambulatory ECG monitor connected to two child‐skin friendly foam electrodes was used to gather cardiac/heart rate data from infants at a sampling frequency of 1000 Hz. We used software from Bittium, the DF viewer software and the Faros Manager software, to extract.edf files of heart rate data (IBIs) after each child was run through the experimental protocol. Next, we extracted HRDSA periods in both Familiarization and Test phases (Figure 1) using algorithms from Neo and Kelleher (2021) that were implemented via *haven*, a software interface designed to streamline visualization and analytics for HRDSA (Kim et al. 2024). For the Familiarization phase, *haven* was used to identify the total number of HRDSA events and the total HRDSA duration for each participant. For the Test phase, we examined the proportion of trials with HRDSA periods between Touch versus No‐Touch trials or between HTP versus LTP trials.

The HPP (Kemler Nelson et al. 1995) was run in a sound booth which housed the three‐sided booth used for the procedure but was controlled from outside the booth via a keypad connected to a computer which ran the Behavioral Infant & Toddler Testing System (or BITTSy; Newman et al. 2021). BITTSy is a free and flexible program that was designed to be able to run the lights and sounds required to administer the HPP for infant testing and to record infant behavior in response to audio and visual displays. The booth itself consisted of three white painted pegboard panels. A light was attached at the center of each panel, at the approximate eye level of an infant seated on a caregiver's lap in the chair. The light on the front panel was green, while the lights on the side panels were both red. A speaker was situated behind each of the two side lights. A video camera which viewed, transmitted a signal to the experimenter outside the booth, and recorded the session was hidden behind the center panel. An ipod which played masking music was connected to the caregiver's Peltor aviation headphones and an additional set of headphones, connected to a splitter and the computer running the procedure, were contained within the booth for the experimenter.

### 2.3 Stimuli

#### 2.3.1 Auditory Stimuli in Familiarization Phase

We constructed 4 Familiarization streams to allow us to counterbalance the different HTP/LTP words and touch/no‐touch items. The counterbalancing of HTP and LTP words were taken directly from Pelucchi et al.’s Experiment 3A/B. Such that *bici* and *casa* were LTP wordforms and *fuga* and *melo* were HTP wordforms in one Familiarization stream, and the reverse was true for a second Familiarization stream. Touch and no‐touch were also counterbalanced, such that in one Familiarization stream *bici* and *fuga* were touch words and in another *casa* and *melo* were touch words. Thus, this full counterbalancing yielded 4 different Familiarization streams/conditions (Table 1).

**TABLE 1:** Word lists used in familiarization and alignment with familiarization conditions.

Condition	Touch‐LTP	No‐touch‐LTP	Touch‐HTP	No‐touch‐HTP
1	bici	casa	fuga	melo
2	casa	bici	melo	fuga
3	fuga	melo	bici	casa
4	melo	fuga	casa	bici

#### 2.3.2 Auditory Stimuli for Infant Testing Phase

Auditory recordings of wordforms tested were identical to those used in Pelucchi et al. Specifically, during each Test trial infants would hear a recording of one of 4 words: *bici, casa, fuga, and melo* played in isolation‐like lists with repetitions of word separated by 500 ms gaps. Playing each word on separate trials allowed us to examine orientation times to HTP/LTP and touch/no‐touch words.

#### 2.3.3 Auditory Stimuli to the Experimenter

In order for experimenters to deliver precisely timed and aligned touches that began exactly at touch‐word onsets and ended precisely with touch‐word offsets, a series of tones was generated to be played on a separate auditory channel from the auditory stimuli to the infant, but that could be precisely aligned with the auditory channel to the infant. We used a channel splitter to ensure that experimenters only heard tones on their headphones. The tone channel played to the experimenter consisted of a brief warning tone to indicate “get ready” to the experimenter, followed by a longer tone aligned with the onset and offset of the target word. Experimenters underwent systematic training to ensure that touches were indeed precisely aligned with target wordforms and were only able to proceed with administering the experimental protocol when 100% of their touches were judged to be well‐aligned and timed. All touches were delivered as gentle squeezes to the infant's knee while the experimenter sat facing the infant in a small chair. Note that this design, in which the statistical learning in Familiarization has a social component, is not all that unusual as caregiver‐child play during a statistical learning Familiarization/Exposure phase has been employed in a variety of other studies (e.g., Estes et al. 2007). Critically, we chose to operationalize touch as a gentle squeeze, since squeezing would be easy for taggers to see on video and is a frequent kind of touch in natural caregiver‐infant interactions (Abu‐Zhaya et al. 2017; Lew‐Williams et al. 2019; Nomikou and Rohlfing 2011). A randomly selected subset of 8 Familiarization videos were hand tagged in ELAN (https://sites.bu.edu/elsa/elan‐coding) to ensure that touches were well‐aligned and timed. This revealed that 100% of touches were well‐aligned (i.e., delivered within 250 ms) with touch words.

### 2.4 Procedure

Infants and caregivers were asked to come into the lab after hearing briefly about the study on the phone or text. Upon arrival in the lab, the procedure was described in detail to caregivers and consent was gathered. After consent was completed, the Bittium Faros heart rate monitor was attached to the chest of the infant under their clothing via two child skin‐friendly electrodes (Kendall foam electrodes) but not turned on. Caregivers were instructed that once they were in the booth they were to act primarily as a “soft seat” for their child and otherwise not interfere with their child's behavior; they were informed that the entire procedure was brief, with each part taking less than 7 minutes (∼21 minutes total).

**FIGURE 2:**
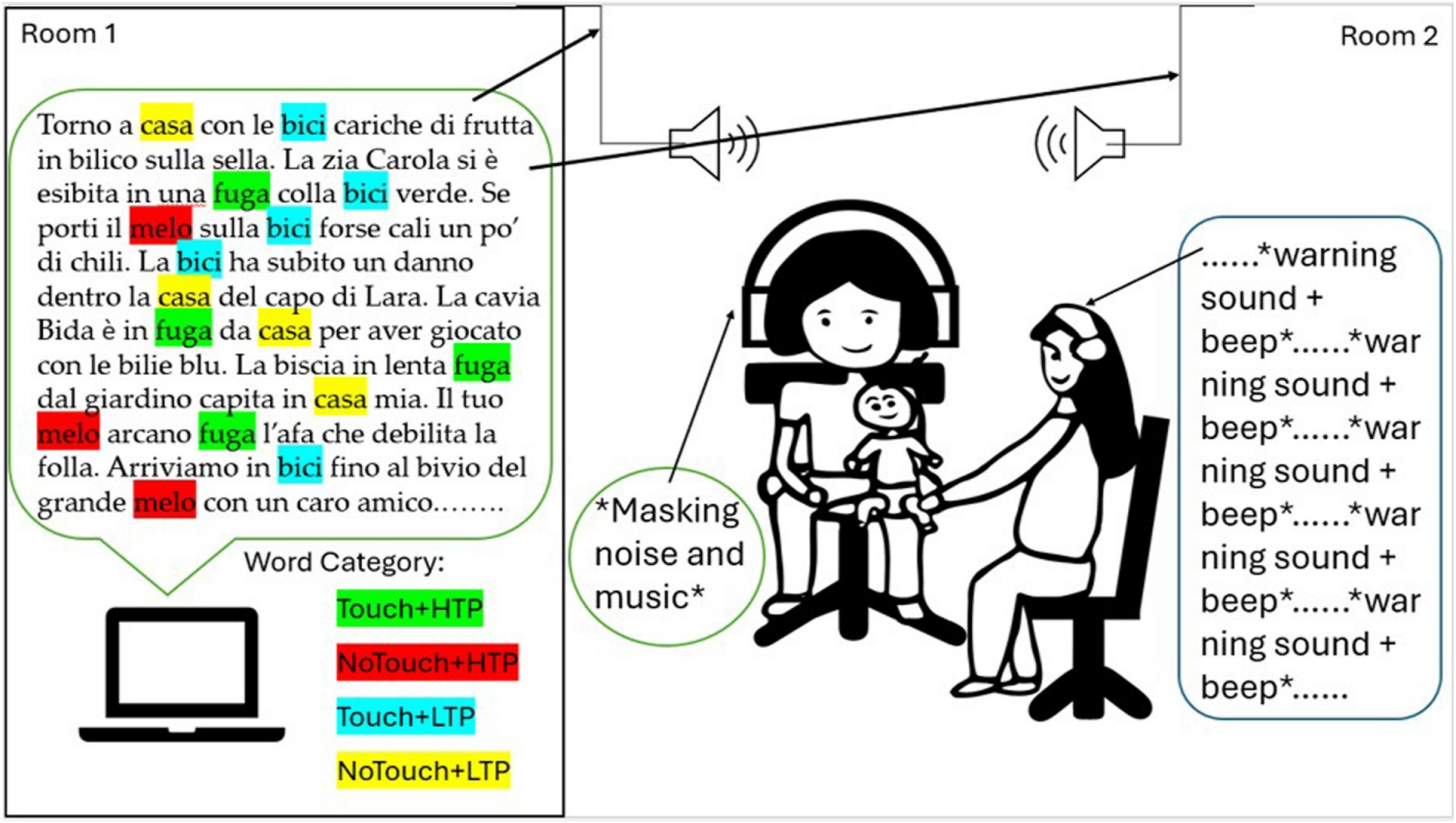
The experiment room setting, with an example of the familiarization stream, showing aligned touch patterns.

After this, the infant and their caregiver were brought into the single‐walled sound booth that housed the HPP, a chair for the caregiver, and a small stool for the experimenter. After seating the infant on their caregiver's lap, a video camera focused on the child and the caregiver was turned on. After this, a dog clicker was clicked at the same time that the Bittium heart rate monitor was turned on. This clicker allowed us to have multiple markers (both auditory and visual) on video, which indicated the time point at which the monitor was turned on to use for later signal alignment. After this, the experimenter placed Peltor aviation headphones on caregivers' ears; these headphones played continuous masking music. The experimenter also placed a set of headphones on her own head. This set of headphones played tone signals. The experimenter then closed the door of the booth and sat down on the small stool facing the infant. Once the first experimenter was seated inside the booth, she gave a thumbs up to a second experimenter seated outside of the booth. This second experimenter was able to view a video of infant, caregiver, and the first experimenter inside the booth. This thumbs up indicated to the second experimenter that she could begin playing the split audio stream. One stream played to two speakers inside the booth (the stream for the infant) and the other stream (tones) played over the first experimenter's headphones. These two auditory streams played for 2 minutes and 14 seconds. After the streams were complete, the first experimenter quickly and quietly left the booth with her stool and gently closed the door. This terminated the Familiarization phase of the experiment and indicated to the second experimenter that it was time to begin the HPP testing (Figure 2).

The second experimenter quickly started the BITTSY (http://langdev.umd.edu/bittsy/) protocol, which caused a center light mounted on the three‐sided panel in the booth to begin flashing. After the experimenter observed, via the video feed from the room, that the infant had oriented toward that light, she pressed a button on a keypad. This caused one of two lights mounted to the right or left of the infant to begin flashing. Once the infant oriented toward the flashing side light, the experimenter pressed another button, which initiated one of the 4 wordlists (corresponding to the 4 words in Table 1) to begin playing. There was a total of 3 blocks of these 4 wordlists and thus a total of 12 experimental trials for each infant. Each auditory word list continued to play until the infant oriented away for more than 2 consecutive seconds, and the program tracked each infant's total orientation time to each word type. The experimenter pressed a button every time the infant orientated away from the side light. After all 12 trials were completed, the experiment was terminated, and the infant and caregiver left the booth. After this, the heart rate monitor was turned off and removed, and caregivers were invited to select a book or toy and collect their infant's scientist certificate.

### 2.5 Data Preparation

#### 2.5.1 Bittsy Data Extraction

Looking time data was extracted from BITTSY's raw data files using the BITTSY Reporting executable script. Orientation times to each word list were recorded, and mean orientation time to each word list type was calculated for each child.

##### 2.5.1.1 Alignment of Heart Rate and Behavioral Data Streams

Since heart rate data was collected during the whole experiment (Familiarization and Test), while the behavioral looking time data was only collected for the Test Phase, and the start and end time of Familiarization Phase and Test Phase were slightly different for each participant, time alignment was necessary. Using the video recordings collected with both the visual clicker and clicker sound, we identified the time points for (1) the start of the Familiarization Phase, (2) the end of the Familiarization Phase, (3) the start of the Test Phase, and (4) the end of the Test Phase for each participant. Using the time points identified, we used an R script to align the start and end times of the Familiarization Phase and Test Phase in heart rate and behavioral looking data streams for each participant (written with the *readxl*, *writexl* and *tidyverse* packages).

#### 2.5.2 Heart‐Rate Data Cleaning

We preprocessed ECG data of the participants following manuals of software EDFBrowser (freeware software by Teuniz van Beelen; Van Beelen 2024) and QRSTool (Allen et al. 2007). See details in Supporting Information S2 (also available on OSF: https://osf.io/z7gya/overview?view_only=e1172d0ee97c48c1a4f9c679808304e5).

#### 2.5.3 Heart Rate Defined Sustained Attention Calculations

We then used the *haven* software mentioned earlier to compute the frequency and duration of HRDSA phases across both Familiarization and Test phases. For each phase, baseline heart rate was defined as the 5 beats preceding the onset of the initial trial stimulus; for Familiarization, this was the onset of the single sound stream; for Test, this was the onset of 1st trial at the light flash. We then identified HRDSA phases, defined as a deceleration in heart rate that exceeded baseline IBI for five consecutive beats. The number and duration of phases were calculated for each participant. This experimental design was not designed to measure HRDSA responses to specific stimuli during the Familiarization period, as inter‐event times ranged from 1 to 4 seconds, and HRDSA responses are registered after 5 consecutive IBIs exceed baseline (e.g., several seconds of data). Instead, HRDSA analyses were used to measure infants' engagement during Familiarization and differential response to stimuli in the Test phase.

## 3 Results

All statistics were run in R and all planned analyses were pre‐registered on OSF (https://osf.io/kvf3c). Analyses were designed to answer the following questions:

1. Do infants weight touch and auditorily presented transitional cues differently in a word segmentation task? Specifically, do infants show different behavioral orientation patterns depending on transitional probability (HTP/LTP) and touch (touch/no‐touch)?
2. Are infants' attentional patterns related to their behavioral patterns? Specifically, do infants who show greater HRDSA also show a greater difference between word types?
3. Is there greater HRDSA to touch words as compared to no‐touch words, as measured during the Test phase of the experiment?

### 3.1 Behavioral Results for HPP Test Phase

We first averaged the orientation/looking times across Test blocks for trials of the same type (i.e., TouchHTP, TouchLTP, NoTouchHTP, or NoTouchLTP) for each participant and then ran the pre‐registered Linear mixed effects regression (LMER) model Orientation time ∼ Word‐type*Touch‐type + (1|Participant) + (1|Language). Linear mixed effects regression (LMER) models are a type of Generalized Linear Mixed Model (GLMM) that assume Gaussian distribution in the data and can be used when the response variable is continuous. In our LMER model, Orientation Time is the continuous response variable, Word‐type, Touch‐type, and the interaction of Word‐type and Touch‐type are the fixed effects (explanatory variables), and Participant and Language are random effects. “Language” (1|Language) refers to the language conditions (3A1, 3A2, 3B1, 3B2) detailed in Table 1.

**FIGURE 3:**
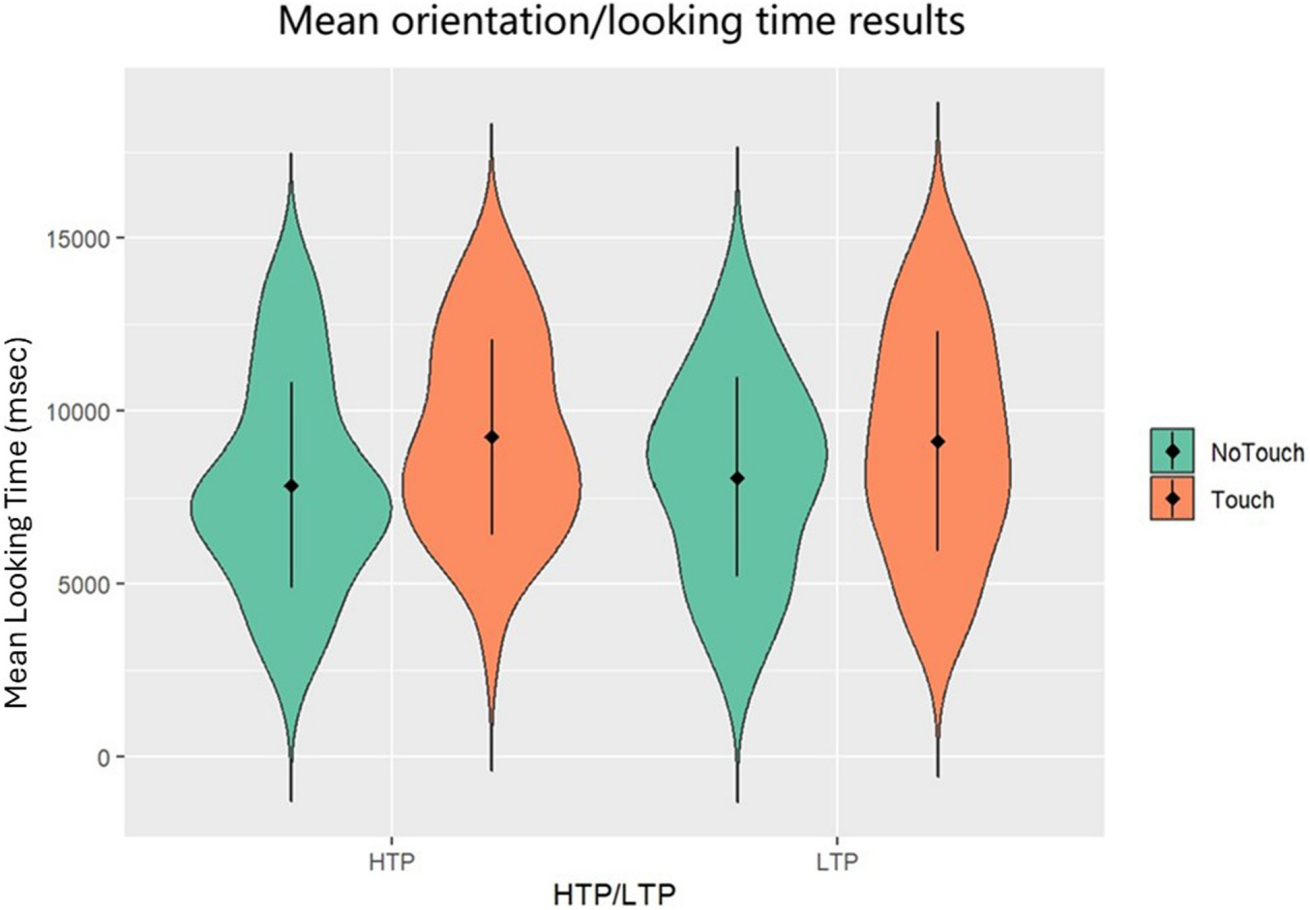
Results of orientation/looking times by trial type.

Results from this model showed that there was a significant main effect of Touch‐type (touch, no‐touch), *F* (1, 108) = 12.1, *β* = 1378.60, *t* (108) = 2.82, *p* < 0.001, while Word‐type (HTP, LTP) and the Word‐type*Touch‐type interaction were not significantly tied to Orientation times, with *F* (1, 108) = 0.03, *β* = 237.30, *t* (108) = 0.491, *p* = 0.86 and *F* (1, 108) = 0.26, *β* = −350.00, *t* (108) = −0.51, *p* = 0.610 respectively. Thus, infants appear to weight touch cues differently than auditory cues about transitional probabilities and spend more time looking at words cued by touch (see Figure 3). Marginal *R* ^*2*^ of the model was 0.041.

### 3.2 Physiological Results for Familiarization Phase

Next, we examined the relationship between HRDSA during Familiarization and behavioral patterns during the Test phase. On average, there were 2.68 periods of heartrate defined sustained attention (HRDSA) per participant in the Familiarization Phase, and the average of total HRDSA duration was 26.90 seconds, with 12 participants having no HRDSA period during the Familiarization Phase (see further details in Supporting Information S1: Appendix Table S1).

**FIGURE 4:**
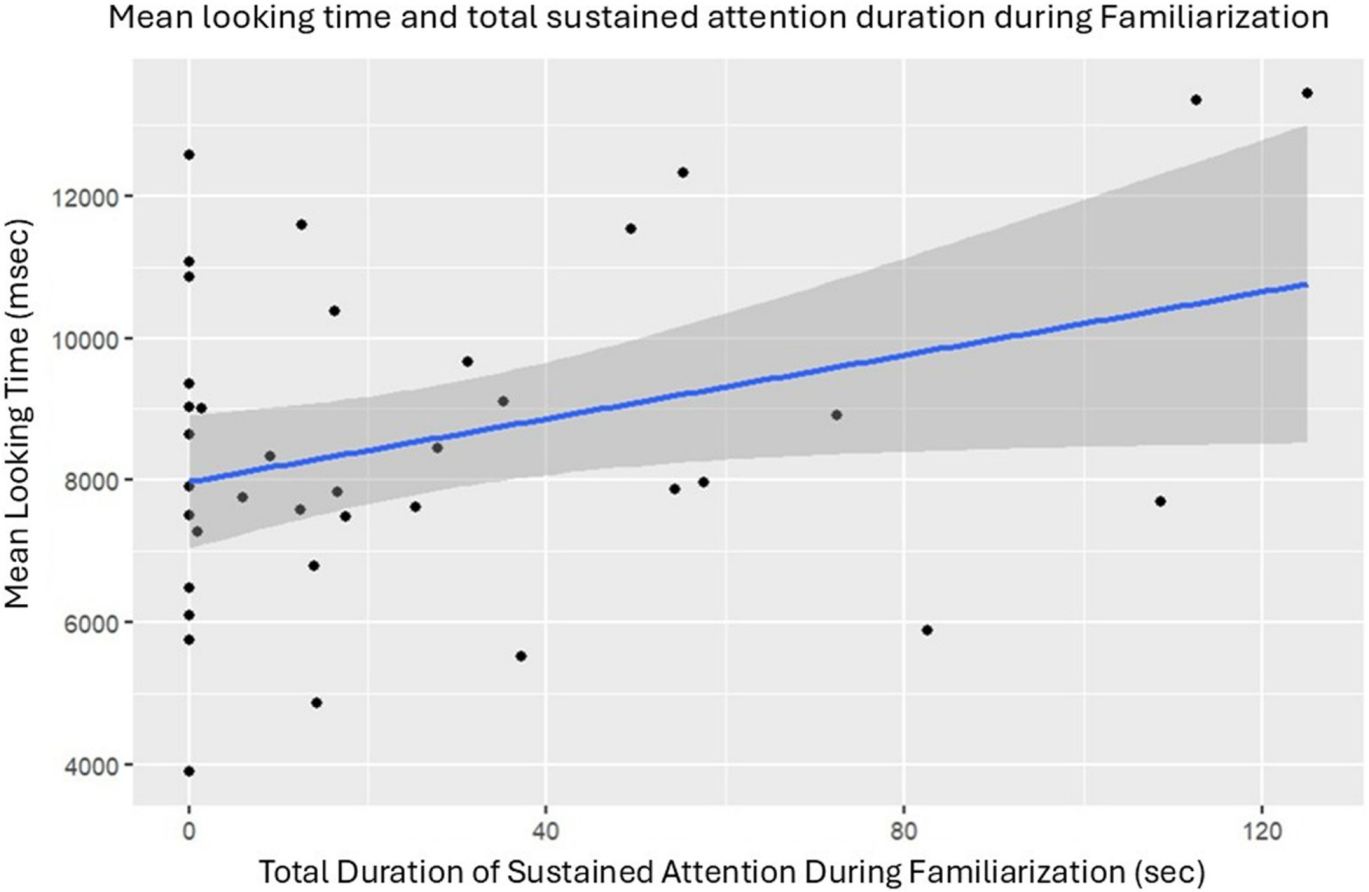
Correlation between total average orientation time during test and the total duration of HRDSA during familiarization.

Because we were curious how overall HRDSA might impact behavioral orientation times, we first examined whether there was a relationship between overall HRDSA during Familiarization and orientation times during the Test phase. Results revealed that there was a significant connection between total HRDSA duration during Familiarization and the total average orientation time (collapsed across orientation times for all word types) at Test, *F* (1, 35) = 4.40, *β* = 22.30, *t* (35) = 2.10, *p* = 0.043, adjusted *R* ^*2*^ = 0.09. These results suggest that infants who showed longer HRDSA during Familiarization also oriented overall longer during the Test Phase (Figure 4). We also examined the relationship between total number of periods of HRDSA during Familiarization and the total average orientation time, but the result here was not significant (*p* = 0.881). Thus, there was a relationship between the duration of HRDSA across HRDSA occurrences, but not the number of unique HRDSA phases, in Familiarization and duration of orienting at Test.

Next, we examined whether the number or duration of HRDSA events in Familiarization was related to performance at Test as measured by the difference score between orientation to familiar versus unfamiliar wordforms. There was no significant relationship between the number or duration of HRDSA events during the Familiarization Phase and the differential average of behavioral orientation time for Touch/No‐Touch and HTP/LTP (means were: TouchHTP = 9.23s; TouchLTP = 9.12s; NoTouchHTP = 7.86s; NoTouchLTP = 8.09s) for each participant, with all *ps* > 0.05 and *F* (1, 35) = 1.89, *β* = 194.40, *t* (35) = 1.38, *F* (1, 35) = 0.00613, *β* = −11.36, *t* (35) = −0.08, *F* (1, 35) = 0.0860, *β* = −3.08, *t* (35) = −0.29, and *F* (1, 35) = 0.381, *β* = 6.46, *t* (35) = 0.62, respectively. In sum, while overall infants who had longer HRDSA during Familiarization showed longer orientation times at Test, this longer orientation and longer HRDSA did not predict differential behavioral responses (orientation) to touch/no‐touch or HTP/LTP wordforms during the Test phase.

### 3.3 Physiological Results for Test Phase

Next, we explored whether, during the Test phase, the likelihood of an infant entering an HRDSA phase differed as a function of word type (HTP, LTP) and touch (Touch, NoTouch). Given the Test phase length was driven by infants' behavioral orientation durations, we excluded Test trials that were shorter than 6 seconds to minimize overlap of physiological HRDSA response (sustained attention of 5 beats beyond a 5‐beat baseline) across trials. We eliminated 11.9% of all trial data based on this 6 second criterion; each participant was required to retain at least 1 trial for each type (TouchHTP, TouchLTP, NoTouchHTP, NoTouchLTP)to be included in analyses. One participant did not meet this threshold, leaving data from only 36 participants to be included in the physiological analyses for the Test phase.

We ran two LMER models to explore the differences in HRDSA duration and number of HRDSA periods by touch or word type. One was HRDSA_duration ∼ Word‐type*Touch‐type + (1|Participant) + (1|Language). In this model, HRDSA_duration is the dependent variable, Word‐type, Touch‐type, and the interaction of Word‐type and Touch‐type are fixed effects, and Participant and Language are random effects. The other was Number of HRDSA periods ∼ Word‐type*Touch‐type + (1|Participant) + (1|Language), with Number of HRDSA periods as the dependent variable, Word‐type and Touch‐type as fixed effects, and Participant and Language as random effects). Results showed that none of the effects were significant, with all *ps* > 0.05, and marginal *R* ^*2s*^ of 0.021 and 0.015 for the two models, respectively (see detailed results in Supporting Information S1: Appendix Table S2). In sum, HRDSA in response to the Test stimuli did not differ based on touch or word type.

## 4 Discussion

Behavioral results were partially in line with our pre‐registered expectations (and previous findings) that aligned touch would have a large impact on word segmentation. However, we predicted that auditorily presented transitional probabilities would, as in previous work (Pelucchi et al. 2009), also impact wordform segmentation such that HTP words with touch would be segmented better than LTP words with touch. Instead, we found that the impact of touch eclipsed the impact of word type (or auditory transitional probability). Why did aligned touch impact auditory wordform segmentation, but auditory TPs fail to impact wordform segmentation in this experiment? First, it is important to note that our auditory stimuli were the same and sample size was similar to that used in the experiments in Pelucchi et al. (2009) paper, so we do not think that differences in these could have impacted our results. Further, while the age range of our children made them overall slightly older than those in Pelucchi et al., analyses with a subsample of our infants with the exact same age range revealed the same pattern as the larger dataset^1^. This means that the only thing that could have contributed to cause differences in our results is the addition of touch cues to our experiment.

There are two hypotheses that might explain our results and why touch had such a strong impact on wordform segmentation here. First, it is possible that aligned touch is special since it is rarer or more unexpected than auditory cues, thus when present, it aids in word segmentation to a greater degree. We can thus think of touches aligned with edges as focusing a flashlight beam on wordform edges while everything else remains murky. This would result in better segmentation around this beam/touch. We term this the *focusing hypothesis*. Second, it is possible that touch simply provides an additional piece of information (in addition to the auditory stream) which serves to support segmentation of wordforms in a challenging task (segmenting from Italian is hard for young English learners). We term this the *information adding* hypothesis. However, if information adding were the *only* or primary benefit of touch, we might expect incremental differences with the addition of touch, so touch might boost LTP wordforms to be segmented as well as HTP wordforms without touch, but that HTP wordforms with touch might be segmented the best of all. Our data seems to provide evidence against this second hypothesis alone as we do not see incremental differences with word type (HTP, LTP) and touch (Touch, NoTouch) –such as both showing additive effects. However, we seem to find strong evidence that touch is special and could serve to focus attention in a uniquely helpful way on constituent edges since the effect of touch on wordform recognition is clearly greater than the effect of transitional probability cues. However, it is still possible that we are simply underpowered to observe such incremental effects due to the information adding hypothesis with this experimental paradigm or that both information adding and focusing are at play here, or that the effects are too small to detect using current design, and larger studies with more trial types and repetitions may be needed. Finally, it is possible that our findings could be limited to the type of touch used here (a gentle squeeze) and that we might have not found the same result had we used a tap or some other kind of touch which was more associated with affect than attention getting such as a gentle pet. Future work can explore whether infants perform differently with different kinds of touches.

One anonymous reviewer also suggested that it is possible that incremental effects for touch and speech cues to edges might have been found had we chosen to use speech cues that were immediately available such as stress cues rather than ones that emerge over time such as TPs. We agree that it is possible that infants relied more heavily on tactile cues than TP cues in our experiment because tactile cues were available immediately, while, given their nature, calculation of TPs occurs across longer strings of speech. Thus, it is possible that if we had used different speech cues that would have been immediately available, such as stress cues or phrasal alignment cues, with tactile cues our results would have been more gradient (e.g., enhancement of stress words with touch, but still differences between stress and non‐stressed words), and children would have showed use of both touch and speech cues to segment the speech stream (e.g., convergence of cue effects). If true, this would suggest that infants do not weight touch over speech cues, just that they rely more heavily on cues which are immediately available. However, it is important to note that prosodic cues seem to show the effects that we predicted (but did not find) for our infants –i.e., convergence of cue effects when combined with TPs. Specifically, often studies show use of *both* prosodic cues and statistical cues together, not that one completely overrides the other (e.g., Shukla et al. 2007; Johnson and Jusczyk 2001; Marimon et al. 2022; Marimon et al. 2025). Thus, we argue that touch may have a unique role in segmentation as compared to any speech cue, including ones that are immediately perceptible.

This brings us back to our first hypothesis: that touch is special and thus we get better segmentation with aligned touch. This hypothesis, however, warrants some nuances. One possibility is that touch is special because when present, it allocates attentional resources more strongly to whatever it is aligned with (in this case a wordform). It is also possible that the special impact of touch does not have anything to do with attention, but rather with affect or something else not measured here. We can only address the former as it was our goal to discern whether touch would impact attentional resources during Familiarization or Test in a way that would lead to differential behavioral results with touch and/or word type.

Critically, although overall duration of HRDSA was associated with overall longer looking at Test regardless of stimulus type, indicating associations between overall attention and looking behaviors, we did *not* find that HRDSA predicted differential learning patterns, as evidenced by behavioral and physiological responses during the Test phase. It is possible that this null finding related to touch and HRDSA reflects that general attentional engagement does not mediate the association between touch and word segmentation, as would be supported by the hypothesis that the special impact of touch is not due to attentional allocation alone. However, given HRDSA is a blunt indicator of general cognitive engagement, with less temporal precision than neural methods such as electroencephalogram (EEG), it is possible that more nuanced aspects of attention driven by touch or fine‐grained linguistic properties, such as novelty detection, may be relevant to learning but not detectable with this specific psychophysiological method or this design. Future designs may include longer inter‐stimulus intervals and more repetitions of learning trials to potentially allow for other heartrate‐related metrics, such as average HR deceleration or arousal in specific time periods, to be collected and analyzed.

Thus, although more work is needed, it appears that we can conclude that while touch did alter behavioral patterns related to wordform segmentation, the effect was not moderated by the amount of HRDSA the infant exhibited during the Test phase. We can only speculate about what mechanisms may support touch specifically aiding in this task and hope to explore this in the future.

## Author Contributions

**Amanda Seidl:** conceptualization, methodology, data curation, investigation, funding acquisition, supervision, formal analysis, project administration, writing – original draft, writing – review and editing. **Boshang Yin:** writing – original draft, writing – review and editing, formal analysis, project administration, data curation. **Bridgette Kelleher:** conceptualization, methodology, software, writing – review and editing, supervision.

## Ethics Statement

The study was conducted in accordance with the ethical standards of the American Psychological Association and was approved by the IRB of Purdue University (IRB project number IRB‐2022‐1471) and the University of Delaware (IRB project number 2079099).

## Conflicts of Interest

The authors declare no conflicts of interest.

## Supporting information


Supporting Information S1

Supporting Information S2

## Data Availability

The data that support the findings of this study are openly available in OSF pre‐registration “Touch to learn: Do tactile cues act as a booster for transitional probabilities?” at https://doi.org/10.17605/OSF.IO/Z7GYA.

## Endnotes

^1^
A subsample of just infants aged 8.0–9.0 within our dataset (*n* = 10) and examined attention to HTP versus LTP words within this sample. Results revealed the same pattern as with the larger sample –a significant difference between Touch versus No Touch (*F* (1, 27) = 5.55, *β* = 1790.70, *t* (27) = 2.06, *p* = 0.049), no significant differences between HTP and LTP wordforms (*F* (1, 27) = 1.73, *β* = 1148.80, *t* (27) = 1.32, *p* = 0.198), and no significant interactions between Touch and TP (*F* (1, 27) = 0.30, *β* = −679.2, *t* (27) = −0.55, *p* = 0.586).

## References

[infa70123-bib-0001] Abu‐Zhaya, R. , A. Seidl , and A. Cristia . 2017. “Multimodal Infant‐Directed Communication: How Caregivers Combine Tactile and Linguistic Cues.” Journal of Child Language 44, no. 5: 1088–1116. 10.1017/S0305000916000416. 27573414

[infa70123-bib-0002] Allen, J. J. B. , A. S. Chambers , and D. N. Towers . 2007. “The Many Metrics of Cardiac Chronotropy: A Pragmatic Primer and a Brief Comparison of Metrics.” Biological Psychology 74, no. 2: 243–262. 10.1016/j.biopsycho.2006.08.005. 17070982

[infa70123-bib-0003] Bahrick, L. , and R. Lickliter . 2002. “Intersensory Redundancy Guides Early Perceptual and Cognitive Development.” In Advances in Child Development and Behavior, edited by R. Kail , 153–187, Academic Press. 10.1016/s0065-2407(02)80041-6 12402674

[infa70123-bib-0004] Bahrick, L. E. , and R. Lickliter . 2000. “Intersensory Redundancy Guides Attentional Selectivity and Perceptual Learning in Infancy.” Developmental Psychology 36, no. 2: 190–201. 10.1037/0012-1649.36.2.190. 10749076 PMC2704001

[infa70123-bib-0005] Bradshaw, J. , X. Fu , and J. E. Richards . 2024. “Infant Sustained Attention Differs by Context and Social Content in the First 2 Years of Life.” Developmental Science 27, no. 4: e13500. 10.1111/desc.13500. 38499474 PMC11608077

[infa70123-bib-0006] Estes, K. G. , J. L. Evans , M. W. Alibali , and J. R. Saffran . 2007. “Can Infants Map Meaning to Newly Segmented Words?: Statistical Segmentation and Word Learning.” Psychological Science 18, no. 3: 254–260. 10.1111/j.1467-9280.2007.01885.x. 17444923 PMC3864753

[infa70123-bib-0007] Feldman, R. , Z. Rosenthal , and A. I. Eidelman . 2014. “Maternal‐Preterm Skin‐to‐Skin Contact Enhances Child Physiologic Organization and Cognitive Control Across the First 10 Years of Life.” Biological Psychiatry 75, no. 1: 56–64. 10.1016/j.biopsych.2013.08.012. 24094511

[infa70123-bib-0008] Feldman, R. , M. Singer , and O. Zagoory . 2010. “Touch Attenuates Infants’ Physiological Reactivity to Stress.” Developmental Science 13, no. 2: 271–278. 10.1111/j.1467-7687.2009.00890.x. 20136923

[infa70123-bib-0009] Ferber, S. G. , R. Feldman , and I. R. Makhoul . 2008. “The Development of Maternal Touch Across the First Year of Life.” Early Human Development 84, no. 6: 363–370. 10.1016/j.earlhumdev.2007.09.019. 17988808

[infa70123-bib-0010] Hollich, G. , R. S. Newman , and P. W. Jusczyk . 2005. “Infants’ Use of Synchronized Visual Information to Separate Streams of Speech.” Child Development 76, no. 3: 598–613. 10.1111/j.1467-8624.2005.00866.x. 15892781

[infa70123-bib-0011] Isbilen, E. S. , and M. H. Christiansen . 2022. “Statistical Learning of Language: A Meta‐Analysis Into 25 Years of Research.” Cognitive Science 46, no. 9: e13198. 10.1111/cogs.13198. 36121309

[infa70123-bib-0012] Jean, A. D. L. , D. M. Stack , and A. Fogel . 2009. “A Longitudinal Investigation of Maternal Touching Across the First 6 Months of Life: Age and Context Effects.” Infant Behavior and Development 32, no. 3: 344–349. 10.1016/j.infbeh.2009.04.005. 19477019 PMC2714702

[infa70123-bib-0013] Johnson, E. K. , and P. W. Jusczyk . 2001. “Word Segmentation by 8‐Month‐Olds: When Speech Cues Count More Than Statistics.” Journal of Memory and Language 44, no. 4: 548–567. 10.1006/jmla.2000.2755.

[infa70123-bib-0014] Johnson, E. K. , A. Seidl , and M. D. Tyler . 2014. “The Edge Factor in Early Word Segmentation: Utterance‐Level Prosody Enables Word Form Extraction by 6‐Month‐Olds.” PLoS One 9, no. 1: e83546. 10.1371/journal.pone.0083546. 24421892 PMC3885442

[infa70123-bib-0015] Jusczyk, P. W. , E. A. Hohne , and A. Bauman . 1999. “Infants’ Sensitivity to Allophonic Cues for Word Segmentation.” Perception & Psychophysics 61, no. 8: 1465–1476. 10.3758/BF03213111. 10598463

[infa70123-bib-0016] Kadlaskar, G. , A. Seidl , H. Tager‐Flusberg , C. A. Nelson , and B. Keehn . 2019. “Atypical Response to Caregiver Touch in Infants at High Risk for Autism Spectrum Disorder.” Journal of Autism and Developmental Disorders 49, no. 7: 2946–2955. 10.1007/s10803-019-04021-0. 31016672 PMC6827711

[infa70123-bib-0017] Kemler Nelson, D. G. , P. W. Jusczyk , D. R. Mandel , J. Myers , A. Turk , and L. Gerken . 1995. “The Head‐Turn Preference Procedure for Testing Auditory Perception.” Infant Behavior and Development 18, no. 1: 111–116. 10.1016/0163-6383(95)90012-8.

[infa70123-bib-0018] Kim, I. L. , L. Zhao , C. Song , W. S. Neo , and B. Kelleher . 2024. “Japper: A Comprehensive Framework for Streamlining Jupyter‐Based Scientific Web Application Development.” In Practice and Experience in Advanced Research Computing 2024: Human Powered Computing, 1–4. 10.1145/3626203.3670594.

[infa70123-bib-0019] Ko, E.‐S. , R. Abu‐Zhaya , E.‐S. Kim , et al. 2023. “Mothers’ Use of Touch Across Infants’ Development and Its Implications for Word Learning: Evidence From Korean Dyadic Interactions.” Infancy 28, no. 3: 597–618. 10.1111/infa.12532. 36757022 PMC10085827

[infa70123-bib-0020] Lew‐Williams, C. , B. Ferguson , R. Abu‐Zhaya , and A. Seidl . 2019. “Social Touch Interacts With Infants’ Learning of Auditory Patterns.” Developmental Cognitive Neuroscience, Social Touch: A New Vista for Developmental Cognitive Neuroscience? 35: 66–74. 10.1016/j.dcn.2017.09.006. PMC5876072 29051028

[infa70123-bib-0021] Longa, L. D. , L. Carnevali , E. Patron , D. Dragovic , and T. Farroni . 2021. “Psychophysiological and Visual Behavioral Responses to Faces Associated With Affective and Non‐Affective Touch in Four‐Month‐Old Infants.” Neuroscience 464: 67–78. 10.1016/j.neuroscience.2020.07.053. 32771501

[infa70123-bib-0022] Marimon, M. , B. Höhle , and A. Langus . 2022. “Pupillary Entrainment Reveals Individual Differences in Cue Weighting in 9‐Month‐Old German‐Learning Infants.” Cognition 224: 105054. 10.1016/j.cognition.2022.105054. 35217262

[infa70123-bib-0023] Marimon, M. , A. Saksida , B. Höhle , and A. Langus . 2025. “Speech Segmentation With Prosodic and Statistical Cues Is Language‐Specific in Infancy.” Languages 10, no. 9: 240. 10.3390/languages10090240.

[infa70123-bib-0024] Neo, W. S. , and B. L. Kelleher . 2021. “Did You Say My Name? Congruency of 12‐Month‐Old Infants’ Behavioral and Cardiac Responses to Name.” Biological Psychology 166: 108207. 10.1016/j.biopsycho.2021.108207. 34662674 PMC8643321

[infa70123-bib-0025] Newman, R. S. , E. A. Shroads , E. K. Johnson , et al. 2021. “Introducing BITTSy: The Behavioral Infant & Toddler Testing System.” Behavior Research Methods 53, no. 6: 2604–2614. 10.3758/s13428-021-01583-9. 34013485

[infa70123-bib-0026] Ngon, C. , A. Martin , E. Dupoux , D. Cabrol , M. Dutat , and S. Peperkamp . 2013. “(Non) Words, (Non) Words, (Non) Words: Evidence for a Protolexicon During the First Year of Life.” Developmental Science 16, no. 1: 24–34. 10.1111/j.1467-7687.2012.01189.x. 23278924

[infa70123-bib-0027] Nomikou, I. , and K. J. Rohlfing . 2011. “Language Does Something: Body Action and Language in Maternal Input to Three‐Month‐Olds.” IEEE Transactions on Autonomous Mental Development 3, no. 2: 113–128. 10.1109/TAMD.2011.2140113.

[infa70123-bib-0028] Pelucchi, B. , J. F. Hay , and J. R. Saffran . 2009. “Statistical Learning in a Natural Language by 8‐Month‐Old Infants.” Child Development 80, no. 3: 674–685. 10.1111/j.1467-8624.2009.01290.x. 19489896 PMC3883431

[infa70123-bib-0029] Richman, A. L. , R. A. LeVine , R. S. New , G. A. Howrigan , B. Welles‐Nystrom , and S. LeVine . 1988. “Maternal Behavior to Infants in Five Cultures.” In Parental Behavior in Diverse Societies, edited by R. A. LeVine , P. M. Miller , and M. M. West , 81–98. Jossey‐Bass. 10.1002/cd.23219884010 3217040

[infa70123-bib-0030] Saffran, J. R. , R. N. Aslin , and E. L. Newport . 1996. “Statistical Learning by 8‐Month‐Old Infants.” Science 274, no. 5294: 1926–1928. 10.1126/science.274.5294.1926. 8943209

[infa70123-bib-0031] Seidl, A. , R. Tincoff , C. Baker , and A. Cristia . 2015. “Why the Body Comes First: Effects of Experimenter Touch on Infants’ Word Finding.” Developmental Science 18, no. 1: 155–164. 10.1111/desc.12182. 24734895

[infa70123-bib-0032] Shukla, M. , M. Nespor , and J. Mehler . 2007. “An Interaction Between Prosody and Statistics in the Segmentation of Fluent Speech.” Cognitive Psychology 54, no. 1: 1–32. 10.1016/j.cogpsych.2006.04.002. 16782083

[infa70123-bib-0033] Stack, D. M. , and D. W. Muir . 1990. “Tactile Stimulation as a Component of Social Interchange: New Interpretations for the Still‐Face Effect.” British Journal of Developmental Psychology 8, no. 2: 131–145. 10.1111/j.2044-835X.1990.tb00828.x.

[infa70123-bib-0034] Stack, D. M. , and D. W. Muir . 1992. “Adult Tactile Stimulation During Face‐to‐Face Interactions Modulates Five‐Month‐Olds’ Affect and Attention.” Child Development 63, no. 6: 1509–1525. 10.2307/1131572. 1446566

[infa70123-bib-0035] Swingley, D. 2005. “Statistical Clustering and the Contents of the Infant Vocabulary.” Cognitive Psychology 50, no. 1: 86–132. 10.1016/j.cogpsych.2004.06.001. 15556130

[infa70123-bib-0036] Thiessen, E. D. , and J. R. Saffran . 2003. “When Cues Collide: Use of Stress and Statistical Cues to Word Boundaries by 7‐ to 9‐Month‐Old Infants.” Developmental Psychology 39, no. 4: 706–716. 10.1037/0012-1649.39.4.706. 12859124

[infa70123-bib-0037] Van Beelen, T. 2024. EDFBrowser (2.12). [Computer Software]. https://www.teuniz.net/edfbrowser/index.html.

[infa70123-bib-0038] Van de Weijer, J. 1998. Language Input for Word Discovery, Vol. 9. Max Planck Institute for Psycholinguistics.

