# Supplementary material for: Touch Supports Word Segmentation More Than Statistics But is Not Mediated by Attention
Source: Infancy. 2026 Aug 27;31(4):e70123. doi: 10.1111/infa.70123 (PMC13521298; doi:10.1111/infa.70123)
Supplement: Supplementary file 1 [file INFA-31-0-s001.pdf]

Heart-rate data cleaning (detailed explanation of procedure)

We translated the ECG data into ASCII format by transforming the raw data files recorded on the Bittium Faros 180 ambulatory ECG monitor of each participant to .txt format with the EDFBrowser (freeware software by Teuniz van Beelen; van Beelen, 2024). This raw-data-to-.txt format transformation did not alter or filter the original data. This translation was necessary for the data to be inputted into QRSTool, a software utilized for heart rate data cleaning and preprocessing for further analyses.

Before importing the data into QRSTool, we removed the ECG signal 20 sec before Familiarization started and after the end of Test phase for the ECG files of all participants. Two researchers then strictly followed the instructions in Allen et al. (2011) on identifying the R spikes. We used the "peri-beat filter" function in QRSTool to expedite the data cleaning process (Allen et al., 2011, p. 4-6). This process involved manually selecting 30 clean sample R spikes, which the peri-beat filter function used to template initial R spike markings for the remaining portion of each file. These initial markings were then manually reviewed for accuracy, and any missed spikes in QRSTool were manually corrected for ECG file of each participant. Then, we used the built-in export function of the QRSTool to extract Beat Times and IBI data (in milliseconds) from the all-R-spikes-identified files. When doing manual corrections, we checked the IBI series (see Figure 3) above the R spikes panel to identify missed R spikes and false R spikes. There was no range of universal values applicable to every participant in the IBI series that we looked for because such a value needs to be dependent on each participant's average HR and contextual factors (e.g., deceleration due to stimuli). Following instructions given by Allen et al. (2011), we used visual inspection as it was often clear to identify missed R waves. We were able to visually check the raw ECG signals and identify the exceptionally long IBI values, typically more than twice the average

IBI value in the local area. For false R waves, the IBI values in those cases were very small (e.g., <50 ms). The built-in export function of QRSTool was then used to extract Beat Times and IBI data in milliseconds as-is from the R-waves and IBI series panels without any further filtering or additional preprocessing steps.

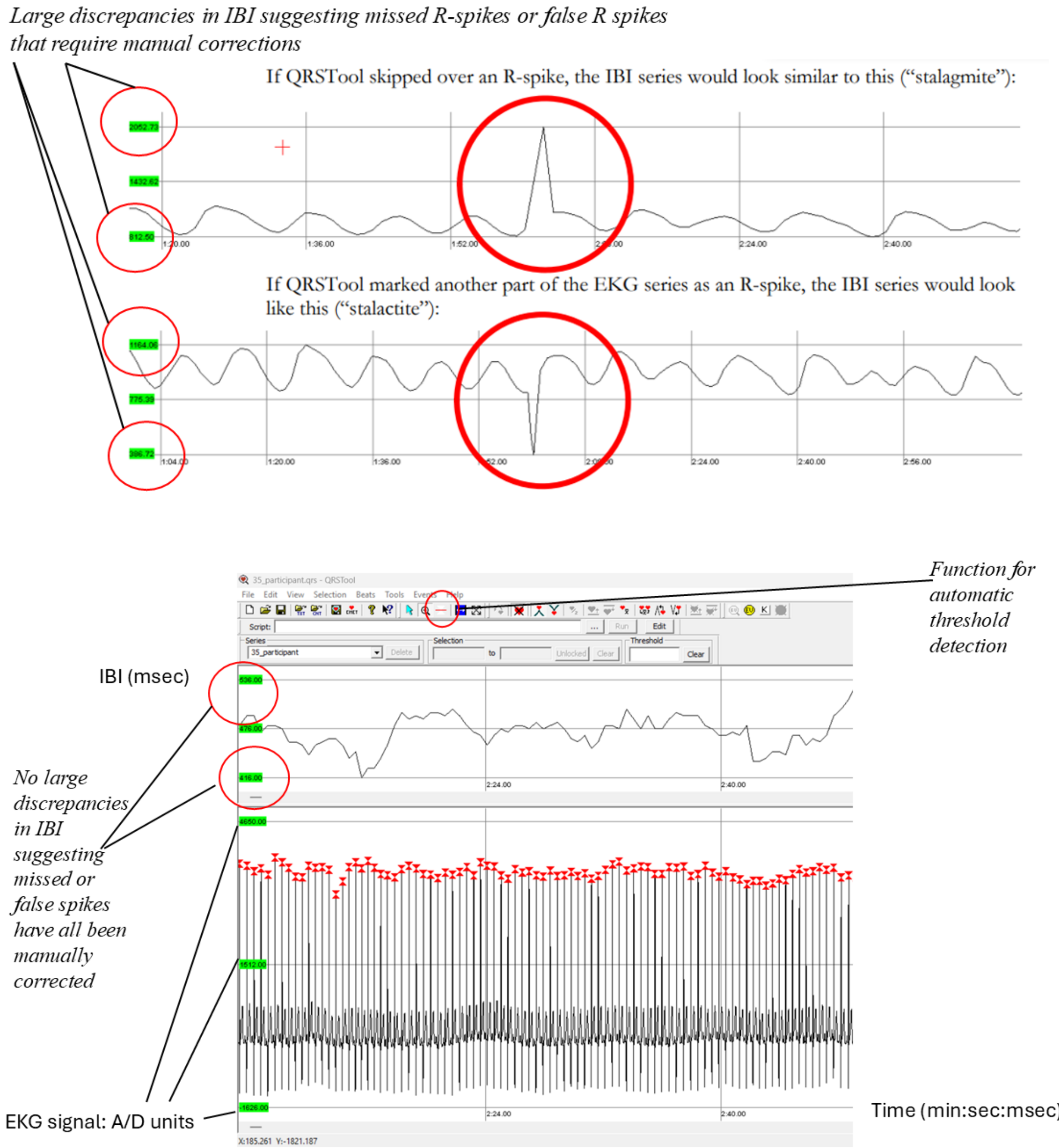

Figure. *Example of data processing in QRSTool with R waves identified.*
