# Supplementary material for: Touch Supports Word Segmentation More Than Statistics But is Not Mediated by Attention
Source: Infancy. 2026 Aug 27;31(4):e70123. doi: 10.1111/infa.70123 (PMC13521298; doi:10.1111/infa.70123)
Supplement: Supplementary file 2 [file INFA-31-0-s002.docx]

**Appendix Table 1**

*Mean number of periods of HRDSA and mean duration of HRDSA during Familiarization*

|  | **Mean Number of Periods of HRDSA During Familiarization** | **Mean Duration of HRDSA During Familiarization** |
| --- | --- | --- |
| **Type** |  |  |
| **NoTouchHTP** | 2.42 | 43.85 |
| **NoTouchLTP** | 2.34 | 43.80 |
| **TouchHTP** | 2.38 | 43.82 |
| **TouchLTP** | 2.27 | 42.13 |

Note. There were in total 10 periods of HRDSA across all participants that did not co-occur with a target word. These periods of HRDSA were included in the calculations of the mean number of periods of HRDSA and mean duration of HRDSA across all participants across all conditions.

**Appendix Table 2**

*HRDSA Test phase results*

|  | **HRDSA Duration** | | | **HRDSA Total Number of Periods** | | |
| --- | --- | --- | --- | --- | --- | --- |
| *Predictors* | *Estimates* | *CI* | *p* | *Estimates* | *CI* | *p* |
| (Intercept) | 2.79 | 2.43 – 3.15 | **<0.001** | 5.00 | 3.45 – 6.55 | **<0.001** |
| Touch type: Touch | 0.03 | -0.46 – 0.52 | 0.898 | -0.71 | -2.91 – 1.48 | 0.511 |
| Transitional Probability: LTP | -0.07 | -0.57 – 0.42 | 0.773 | -0.12 | -2.25 – 2.00 | 0.905 |
| Touch type × Transitional Probability | -0.34 | -1.03 – 0.35 | 0.330 | 0.51 | -2.39 – 3.40 | 0.723 |
| **Random Effects** | | | | | | |
| σ^2^ | 1.25 | | | 4.03 | | |
| τ_00_ | 0.00 _Participant_ | | | 0.00 _Condition_ | | |
|  | 0.00 _Condition_ | | |  | | |
| N | 35 _Participant_ | | | 4 _Condition_ | | |
|  | 4 _Condition_ | | |  | | |
| Observations | 164 | | | 34 | | |
| Marginal R^2^ / Conditional R^2^ | 0.021 / NA | | | 0.015 / NA | | |
